# Routine Vaccination Coverage — Worldwide, 2019

**DOI:** 10.15585/mmwr.mm6945a7

**Published:** 2020-11-13

**Authors:** Anna N. Chard, Marta Gacic-Dobo, Mamadou S. Diallo, Samir V. Sodha, Aaron S. Wallace

**Affiliations:** ^1^Epidemic Intelligence Service, CDC; ^2^Global Immunization Division, Center for Global Health, CDC; ^3^Department of Immunization, Vaccines and Biologicals, World Health Organization, Geneva, Switzerland; ^4^Division of Data, Research and Policy, United Nations Children’s Fund, New York, New York.

Endorsed by the World Health Assembly in 2020, the Immunization Agenda 2030 strives to reduce morbidity and mortality from vaccine-preventable diseases across the life course (*1*). This report, which updates previous reports (*2*), presents global, regional,* and national vaccination coverage estimates and trends as of 2019 and describes the number of surviving infants who did not receive the first dose of diphtheria and tetanus toxoids and pertussis-containing vaccine (DTP1) during the first year of life (i.e., zero-dose children), which serves as a proxy for children with poor access to immunization and other health services. Global estimates of coverage with the third dose of DTP (DTP3), the first dose of measles-containing vaccine (MCV1), and the third dose of polio vaccine (Pol3) ranged from 84% to 86% during 2010–2019. Worldwide, 19.7 million children (15%) were not vaccinated with DTP3 in 2019, 13.8 million (70%) of whom were zero-dose children. During 2010–2019, the number of zero-dose children increased in the African, Americas, and Western Pacific regions. Global coverage with the second MCV dose (MCV2) increased from 42% in 2010 to 71% in 2019. During 2010–2019, global coverage with underused vaccines increased for the completed series of rotavirus vaccine (rota), pneumococcal conjugate vaccine (PCV), rubella-containing vaccine (RCV), *Haemophilus influenzae* type b vaccine (Hib), hepatitis B vaccine (HepB), and human papillomavirus vaccine (HPV). Achieving universal coverage with all recommended vaccines will require tailored, context-specific strategies to reach communities with substantial proportions of zero-dose and incompletely vaccinated children, particularly those in remote rural, urban poor, and conflict-affected communities (*3*).

In 1974, the World Health Organization (WHO) established the Expanded Programme on Immunization to ensure that all infants have access to four recommended vaccines (bacillus Calmette-Guérin vaccine [BCG], DTP, Pol, and MCV) to protect against six diseases (tuberculosis, diphtheria, tetanus, pertussis, poliomyelitis, and measles). Since then, additional vaccines and doses have been introduced in the first year of life (PCV, rota, RCV, Hib, and HepB) and beyond (MCV2 and HPV) (*4*). WHO and the United Nations Children’s Fund (UNICEF) derive national vaccination coverage estimates through annual country-by-country review of available data, including administrative^†^ and survey-based coverage (*5*,*6*); generally, only doses administered through routine immunization visits are counted. DTP3 coverage by age 12 months is considered an indicator of immunization program performance. Children who have not received any doses of DTP by age 12 months (zero-dose children) represent a lack of access to immunization services; those who receive DTP1 but do not complete the series are considered to have dropped out. DTP1-to-DTP3 dropout, an indicator of immunization program utilization, is calculated as the percentage of children who received DTP1 but not DTP3.

Based on WHO and UNICEF estimates during 2010–2019, global coverage with DTP1 (89%–90%) and DTP3 (84%–85%) remained stable. The only region with a decline in DTP3 coverage during 2000–2019 was the Americas (from 91% to 84%). In 2019, DTP1 coverage ranged from 81% in the African region to 97% in the European region (Table 1). DTP3 coverage followed similar regional trends, with estimates ranging from 74% in the African region to 95% in the European region. Among 19.7 million children worldwide who did not complete the 3-dose DTP series in 2019, 13.8 million (70%) were zero-dose children and 5.9 million (30%) had started, but not completed, the DTP series. In 2019, overall DTP1-to-DTP3 dropout was 6% and ranged from 1% in the Western Pacific region to 9% in the African region.

**TABLE 1:** Vaccination coverage, by vaccine and World Health Organization (WHO) region — worldwide, 2019

Vaccine	No. (%) of countries with vaccine in schedule	WHO region % coverage*						
		Global	AFR	AMR	EMR	EUR	SEAR	WPR
BCG	156 (80)	88	80	83	87	92	93	96
DTP1	194 (100)	90	81	90	89	97	94	95
DTP3	194 (100)	85	74	84	82	95	91	94
HepB BD	111 (49)	43	6	55	34	41	54	84
HepB3	189 (97)	85	73	81	82	92	91	94
Hib3	192 (98)	72	73	85	82	79	89	24
HPV, last^†^	106 (55)	15	19	55	0	24	2	4
MCV1	194 (100)	85	69	88	82	96	94	94
MCV2	178 (91)	71	33	75	75	91	83	91
PCV3	148 (74)	48	70	83	52	80	23	14
Pol3	194 (100)	86	74	87	83	95	90	94
RCV1	173 (88)	71	33	88	45	96	93	94
Rota, last^§^	108 (52)	39	50	74	49	25	37	2

**Abbreviations:** AFR = African Region; AMR = Region of the Americas; BCG = bacille Calmette-Guérin vaccine; DTP1 = first dose of diphtheria and tetanus toxoids and pertussis-containing vaccine; DTP3 = third dose of diphtheria and tetanus toxoids and pertussis-containing vaccine; EMR = Eastern Mediterranean Region; EUR = European Region; HepB BD = birth dose of hepatitis B vaccine; HepB3 = third dose of hepatitis B vaccine; Hib3 = third dose of *Haemophilus influenzae* type b vaccine; HPV, last = final dose of human papillomavirus vaccine; MCV1 = first dose of measles-containing vaccine; MCV2 = second dose of measles-containing vaccine; PCV3 = third dose of pneumococcal conjugate vaccine; Pol3 = third dose of polio vaccine; RCV1 = first dose of rubella-containing vaccine; Rota, last = final dose of rotavirus vaccine series; SEAR = South-East Asia Region; WPR = Western Pacific Region.

* BCG coverage is based on 156 countries with BCG in the national schedule, whereas coverage for all other vaccines is based on 194 countries (global) or all countries in the specified region. Administrative coverage is the number of vaccine doses administered to those in a specified target group divided by the estimated target population. During vaccination coverage surveys, a representative sample of households are visited and caregivers of children in a specified target group (e.g., aged 12–23 months) are interviewed. Dates of vaccination are transcribed from the child's home-based record, recorded based on caregiver recall, or transcribed from health facility records. Survey-based vaccination coverage is calculated as the proportion of persons in a target age group who received a vaccine dose.

^†^ Number of doses to complete the HPV series depends on age of recipient.

^§^ Number of doses to complete the rota series varies among vaccine products.

The number of zero-dose children varied by region and economic classification^§^ (Table 2). The number of zero-dose children changed little or declined in all regions from 2000 to 2010. However, during 2010–2019, the number of zero-dose children increased in the African region (from 6.1 million to 6.8 million), the Americas (from 0.5 million to 1.5 million), and the Western Pacific region (from 0.9 million to 1.2 million).

**TABLE 2:** Number of surviving infants not receiving DTP1 (zero-dose children), by World Health Organization (WHO) region and World Bank economic classification — worldwide, 2000–2019

Characteristic/Year	WHO region							Economic classification*		
	Global	AFR	AMR	EMR	EUR	SEAR	WPR	Low	Middle	High
**2000**										
Total no. of countries	191	46	35	21	52	10	27	63	86	37
No. of surviving infants (millions)	124.6	24.1	15.5	13.8	10.1	37.3	24.0	69.8	44.5	10.2
Global % of surviving infants	—	19	12	11	8	30	19	56	36	8
No. of zero-dose children (millions)	21.4	8.2	0.5	2.7	0.3	8.2	1.5	18.9	2.2	0.3
Global % of zero-dose children	—	38	2	13	1	38	7	88	10	1
**2010**										
Total no. of countries	193	46	35	21	53	11	27	35	106	49
No. of surviving infants (millions)	133.0	30.5	15.0	16.1	11.2	35.8	24.4	25.1	95.3	12.6
Global % of surviving infants	–	23	11	12	8	27	18	19	72	9
No. of zero-dose children (millions)	14.9	6.1	0.5	2.6	0.5	4.3	0.9	3.6	11.0	0.3
Global % of zero-dose children	—	41	3	17	3	29	6	24	74	2
**2019**										
Total no. of countries	194	47	35	21	53	11	27	29	103	60
No. surviving of infants (millions)	135.6	35.8	14.6	17.3	10.9	33.8	23.2	21.8	101.3	12.5
Global % of surviving infants	—	26	11	13	8	25	17	16	75	9
No. of zero-dose children (millions)	13.8	6.8	1.5	2.0	0.3	2.0	1.2	4.0	9.5	0.3
Global % of zero-dose children	—	49	11	14	2	14	9	29	69	2

**Abbreviations:** AFR = African Region; AMR = Region of the Americas; DTP1 = first dose of diphtheria and tetanus toxoids and pertussis-containing vaccine; EMR = Eastern Mediterranean Region; EUR = European Region; SEAR = South-East Asia Region; WPR = Western Pacific Region.

* Low-income economies are defined as those with a gross national income (GNI), in USD, per capita in 2000 of ≤$755, in 2010 of ≤$1,005, and in 2019 of ≤$1,035; middle-income economies are those with a GNI per capita in 2000 of $756–$9,265, in 2010 of $1,006–$12,275, and in 2019 of $1,036–$12,535; high-income economies are those with a GNI per capita in 2000 of >$9,265, in 2010 of >$12,275, and in 2019 of >$12,535; calculated using the World Bank Atlas method (https://datahelpdesk.worldbank.org/knowledgebase/articles/906519-world-bank-country-and-lending-groups). Cook Islands and Niue, in the Western Pacific Region, are missing GNI data and are excluded from this categorization.

In 2000, low-income countries accounted for the highest percentage of zero-dose children (88%; 18.9 million); by 2019, however, middle-income countries accounted for the highest percentage of zero-dose children (69%; 9.5 million). This shift occurred largely because 36 countries advanced from low- to middle-income classification from 2000 to 2019 and because the number of zero-dose children increased in 32 (51%) of the 63 countries classified as middle-income in both 2000 and 2019. In 2019, 10.6 million (77%) zero-dose children lived in countries eligible for support from Gavi, the Vaccine Alliance^¶^; these countries receive financial assistance to pay for vaccines and health system strengthening to extend the reach and quality of their immunization programs. Approximately two thirds (65%; 9.0 million) of zero-dose children in 2019 lived in 10 countries: Nigeria, India, Democratic Republic of the Congo (DRC), Pakistan, Ethiopia, Brazil, Philippines, Indonesia, Angola, and Mexico (Figure). Fragile or conflict-affected countries** accounted for 44% of zero-dose children in 2019.

**FIGURE:**
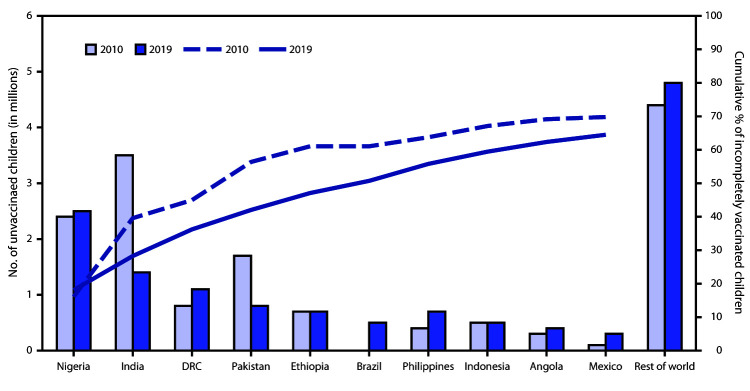
Estimated number of zero-dose children* among the 10 countries with the most zero-dose children and cumulative percentage of all incompletely vaccinated children accounted for by these 10 countries — worldwide, 2019* **Abbreviation:** DRC = Democratic Republic of the Congo. * Zero-dose children are surviving infants who did not receive the first dose of diphtheria and tetanus toxoids and pertussis-containing vaccine during the first year of life.

During 2010–2019, global coverage with MCV1 remained stable at 84%–85%, and in 2019 ranged from 69% in the African region to 96% in the European region. MCV2 coverage increased from 42% to 71% (Table 1). Among all countries (including those yet to introduce MCV2), coverage ranged from 33% in the African region to 91% in the European and Western Pacific regions. Among underused vaccines, global coverage increased during 2010–2019 for the completed series of rota (from 8% to 39%), PCV (from 11% to 48%), RCV (first dose: from 35% to 71%), Hib (from 40% to 72%), HepB (birth dose: from 26% to 43%; 3-dose series: from 73% to 85%), and HPV (from 3% to 15%) (Table 1).

## Discussion

Since establishment of the Expanded Programme on Immunization in 1974, substantial progress in vaccination coverage has been made worldwide. In 2019, 90% of children received at least 1 DTP dose and 85% received 3 DTP doses and at least 1 MCV dose. However, challenges to achieving higher routine immunization coverage remain. Despite large gains in vaccination coverage during 2000–2010, coverage with established vaccines has increased little since 2010 and progress is uneven: coverage in the African region lags that in other regions, and progress in the Americas has reversed.

Extending immunization services to regularly reach zero-dose and underimmunized children and communities is one of the objectives of the Immunization Agenda 2030 (*1*). Low-income, fragile, and conflict-affected countries are homes to large numbers of zero-dose children and remain vulnerable to outbreaks of vaccine-preventable diseases. Since 2010, however, a larger proportion of zero-dose children live in middle-income countries. Although some middle-income countries experienced notable declines in DTP1 coverage (e.g., Brazil, Mexico, Philippines), this shift is driven mostly by countries advancing from low-income to middle-income status. As countries’ economic statuses advance, they become less eligible for external funding, necessitating increasing domestic investments in immunization programs. Identifying demographic, social, and systemic factors inhibiting vaccine delivery and developing locally tailored, context-specific strategies to increase access, availability, and demand for immunization services will be important for reaching zero-dose children. Increasing and optimizing vaccine delivery opportunities at existing health system contact points can reduce missed vaccination opportunities (*7*); providing catch-up vaccination, particularly for older children who missed doses, can help close coverage gaps that would otherwise grow as populations age.

Catch-up policies and strategies will be essential to recovering from disruptions to routine immunization programs experienced during the coronavirus disease 2019 (COVID-19) pandemic. Although countries have attempted to maintain their immunization programs, reduced availability of health workers and personal protective equipment, vaccine distribution system delays, and reduced demand for immunization have contributed to fewer children being vaccinated in 2020 (*8*,*9*). Addressing immunization gaps created by the pandemic will require monitoring immunization program setbacks, implementing catch-up vaccination policies and strategies, and expanding and intensifying routine immunization services.

The findings in this report are subject to at least three limitations. First, data quality limitations could have resulted in inaccurate estimations of administrative coverage. Second, recall bias could have affected survey-based estimates of coverage (*5*). Finally, conflict-affected countries likely have limited external evaluation of coverage, which might have affected accuracy of coverage estimates.

Increasing vaccination coverage above the levels achieved in the past decade will require locally driven, targeted strategies that address barriers to vaccination, particularly in communities with large populations of zero-dose children. Reducing missed opportunities for vaccination and defining country-specific strategies for catch-up vaccination, especially during the COVID-19 pandemic, can improve vaccination coverage and help advance progress toward achieving global immunization goals.

**Summary**

**What is already known about this topic?**

Global coverage with the third dose of diphtheria and tetanus toxoids and pertussis-containing vaccine (DTP), third dose of polio vaccine, and first dose of measles-containing vaccine has remained between 84% and 86% since 2010.

**What is added by this report?**

In 2019, 13.8 million children worldwide did not receive the first dose of DTP (zero-dose children). During 2010–2019, the number of zero-dose children increased in the African, Americas, and Western Pacific regions.

**What are the implications for public health practice?**

Increasing vaccination coverage beyond levels achieved in the past decade will require targeted, context-specific strategies to identify zero-dose and underimmunized children, introduce interventions to minimize missed vaccinations, monitor vaccination coverage, and respond to immunization program setbacks.

## Footnotes

* Based on World Health Organization regional classifications. https://www.who.int/about/who-we-are/regional-offices.

† For a given vaccine, the administrative coverage is the number of vaccine doses administered to persons in a specified target group divided by the estimated target population. Doses administered during routine immunization visits are counted, but doses administered during supplemental immunization activities (mass campaigns) usually are not. During vaccination coverage surveys, a representative sample of households is visited, and caregivers of children in a specified target age group (e.g., aged 12–23 months) are interviewed. Dates of vaccination are transcribed from the child’s home-based record, recorded based on caregiver recall, or transcribed from health facility records. Survey-based vaccination coverage is calculated as the proportion of persons in a target age group who received a vaccine dose.

§ Low-income economies are defined as those with a gross national income (GNI), in USD, per capita in 2000 of ≤$755, in 2010 of ≤$1,005, and in 2019 of ≤$1,035; middle-income economies are those with a GNI per capita in 2000 of $756–$9,265, in 2010 of $1,006–$12,275, and in 2019 of $1,036–$12,535; high-income economies are those with a GNI per capita in 2000 of >$9,265, in 2010 of >$12,275, and in 2019 of >$12,535; calculated using the World Bank Atlas method (https://datahelpdesk.worldbank.org/knowledgebase/articles/906519-world-bank-country-and-lending-groups). Cook Islands and Niue (Western Pacific Region) are missing GNI data and are excluded from this categorization.

¶ Based on Gavi 4.0, eligibility includes 68 low- and middle-income countries eligible to receive financial assistance through grants contingent on a country’s GNI per capita. Eligibility is defined as a country’s average 3-year GNI per capita in USD of ≤$1,580. As GNI increases, a country moves through Gavi’s different eligibility phases until reaching the transition phase when GNI exceeds the eligibility threshold. https://www.gavi.org.

** Based on the World Bank’s classification of fragile and conflict-affected situations for 2019. Fragile countries are defined as those with high levels of institutional and social fragility, measured by the quality of policy, institutions, and manifestations of fragility. Conflict-affected countries are defined as those affected by violent conflict, measured by the number of conflict-related deaths per capita. https://www.worldbank.org/en/topic/fragilityconflictviolence/brief/harmonized-list-of-fragile-situations.
